# Profiling of kidney involvement in systemic lupus erythematosus by deep learning using the National Database of Designated Incurable Diseases of Japan

**DOI:** 10.1007/s10157-023-02337-x

**Published:** 2023-03-16

**Authors:** Tomonori Kimura, Hidekazu Ikeuchi, Mitsuaki Yoshino, Ryuichi Sakate, Shoichi Maruyama, Ichiei Narita, Keiju Hiromura

**Affiliations:** 1 Reverse Translational Research Project, Center for Rare Disease Research, National Institutes of Biomedical Innovation, Health and Nutrition (NIBIOHN), Ibaraki, Osaka Japan; 2 Laboratory of Rare Disease Resource Library, Center for Rare Disease Research, National Institutes of Biomedical Innovation, Health and Nutrition (NIBIOHN), Ibaraki, Osaka Japan; 3 Department of Nephrology and Rheumatology, Gunma University Graduate School of Medicine, Maebashi, Gunma Japan; 4 Department of Nephrology, Nagoya University Graduate School of Medicine, Nagoya, Aichi Japan; 5 Division of Clinical Nephrology and Rheumatology, Kidney Research Center, Niigata University Graduate School of Medical and Dental Sciences, Niigata, Japan

**Keywords:** Systemic lupus erythematosus, Kidney involvement, Profiling, Deep learning, National database, Designated incurable diseases

## Abstract

**Background:**

Kidney involvement frequently occurs in systemic lupus erythematosus (SLE), and its clinical manifestations are complicated. We profiled kidney involvement in SLE patients using deep learning based on data from the National Database of Designated Incurable Diseases of Japan.

**Methods:**

We analyzed the cross-sectional data of 1655 patients with SLE whose Personal Clinical Records were newly registered between 2015 and 2017. We trained an artificial neural network using clinical data, and the extracted characteristics were evaluated using an autoencoder. We tested the difference of population proportions to analyze the correlation between the presence or absence of kidney involvement and that of other clinical manifestations.

**Results:**

Data of patients with SLE were compressed in a feature space in which the anti-double-stranded deoxyribonucleic acid (anti-dsDNA) antibody titer, antinuclear antibody titer, or white blood cell count contributed significantly to distinguishing patients. Many SLE manifestations were accompanied by kidney involvement, whereas in a subgroup of patients with high anti-dsDNA antibody titers and low antinuclear antibody titers, kidney involvement was positively and negatively correlated with hemolytic anemia and inflammatory manifestations, respectively.

**Conclusion:**

Although there are various combinations of SLE manifestations, our study revealed that some of them are specific to kidney involvement. SLE profiles extracted from the objective analysis will be useful for categorizing SLE manifestations.

**Supplementary Information:**

The online version contains supplementary material available at 10.1007/s10157-023-02337-x.

## Introduction

Systemic lupus erythematosus (SLE) is accompanied by various symptoms and complications, often including kidney involvement [1]. In the context of SLE, kidney involvement is caused by various pathogenetic mechanisms, and most often manifests as lupus nephritis. Lupus nephritis is characterized by inflammatory lesions, primarily in the glomerulus, that are caused by complement activation, inflammatory cell infiltration, and the deposition of immune complexes such as those consisting of anti-double-stranded deoxyribonucleic acid (anti-dsDNA) antibodies and DNA[2]. Lupus nephritis is histologically diverse and currently classified into six classes according to the International Society of Nephrology/Renal Pathology Society Classification [3]. Besides lupus nephritis, thrombotic lesions associated with antiphospholipid antibodies and thrombotic microangiopathy may also lead to kidney involvement in SLE patients [4, 5]. The kidney prognosis of SLE patients is improving in Japan [6], while profiling of kidney involvement is necessary for further improvement.

Without appropriate treatment, kidney involvement in SLE patients may lead to end-stage kidney disease and even death [7, 8]. It is important to understand and categorize the various manifestations of kidney involvement in SLE, both to improve outcomes and to identify molecular targets of treatment. This requires patient stratification to create homogeneous groups for research.

Recently, machine learning has started to be used in the stratification of intractable diseases. Deep learning is an artificial intelligence technology that helps unravel potential patterns in multiple factors. Deep learning methods are being applied in observational studies to analyze clinical patterns of diseases based on complex combinations of clinical parameters. The use of an autoencoder (self-encoder), an unsupervised machine learning method, is useful for learning objectively without bias, even in a small number of cases [9–12]. Employing a combination of deep learning methods may help characterize SLE subgroups.

The National Database of Designated Incurable Diseases of Japan (NDDID-J) was established in Japan, and consists of data from the Personal Clinical Records (PCRs) submitted when individuals apply for medical support. These PCRs contain clinically significant information such as symptoms, laboratory test results, and histopathological findings. This database is suitable for deep learning since it contains data from a large number of patients throughout Japan, and is expected to be used to elucidate the pathophysiology of intractable diseases.

In this study, we mathematically profiled SLE-related kidney involvement using deep learning that employed the data from NDDID-J. We evaluated features of kidney involvement mapped on a feature space of SLE that was created by machine learning without bias. We also explored subgroups of patients with SLE-related kidney involvement who exhibited distinct clinical complications. Using machine learning, this study revealed different types of kidney involvement in SLE patients.

## Methods

### Intractable disease database

In a collaborative research group on intractable kidney involvement, we have been conducting a project to elucidate various manifestations of kidney involvement based on data from NDDID-J using deep learning and cluster analysis by artificial intelligence. There are six prerequisites for designating a disease as an intractable disease: (1) the cause is unknown, (2) no effective treatment has been established, (3) the disease is rare, (4) the disease has a chronic course, (5) the number of patients in Japan does not exceed a specified number (approximately 0.1% of the national population), and (6) objective diagnostic criteria have been established. The medical care benefit system for patients with designated intractable diseases facilitates research (i.e., epidemiological, clinical, and that related to drug discovery) because data from individuals who apply for medical support are accumulated in NDDID-J. Since the Act on Medical Care for Patients with Intractable Diseases was enforced in 2015, data on 338 diseases (as of September 2022) collected from PCRs have been added to the database.

This study analyzed data from PCRs of SLE patients that were newly registered in the database between January 2015 and March 2018. The study protocol was approved by the ethics committee of the Japanse Society of Nephrology (approval number #70), the institutional review board of each participating hospital, and the working group on data provision of the Ministry of Health, Labour and Welfare. The reported statistics herein were generated by the users of NDDID-J and were different from those generated and reported by the Ministry of Health, Labour and Welfare. Written informed consent from patients was obtained to register PCR data in the database and to use the data for research and policy-making. All procedures performed in the present study were in accordance with the Declaration of Helsinki.

### Data characteristics and curation

The 75 survey items on the PCR used for the new registration of SLE are shown in Table S1. These items are categorized into clinical manifestations, laboratory test results, and autoantibodies. Unmodifiable factors, such as age and sex, were excluded from the analysis, as we considered that correlations within modifiable variables and correlations between modifiable and unmodifiable variables cannot be evaluated in the same way. We aimed to elucidate the pathophysiology, and drug information was also excluded because of the complexity. Data on clinical manifestations were classified as (1) present, (2) absent, or (3) unknown. Laboratory test data are presented as continuous values. For autoantibodies, data on antinuclear antibodies and anti-dsDNA antibodies are presented as titers, and data on other autoantibodies are presented as (1) positive, (2) negative, or (3) unknown. The presence or absence of kidney involvement was defined using data recorded in the PCR.

Kidney involvement was considered to be absent if each of the following criteria was met:

- Urine protein qualitative test: negative;
- Urine protein per day: < 0.5 g or no data;
- Granular casts: not observed;
- Renal/urological clinical manifestations (i.e., rapidly progressive glomerulonephritis, nephrotic syndrome, acute renal failure, chronic renal failure): not observed.

Data used in machine learning had to be processed and cleaned in advance to make them suitable for analysis. If multiple patients had identical data, the data of one patient were included in the analysis and the duplicate data were excluded. Erroneous data (e.g., values that were entered although no tests were performed) were included in the analysis after likely values were assigned as alternatives. These likely values were determined according to the basic statistics of the data.

### Unsupervised machine learning classifier

**Fig. 1:**
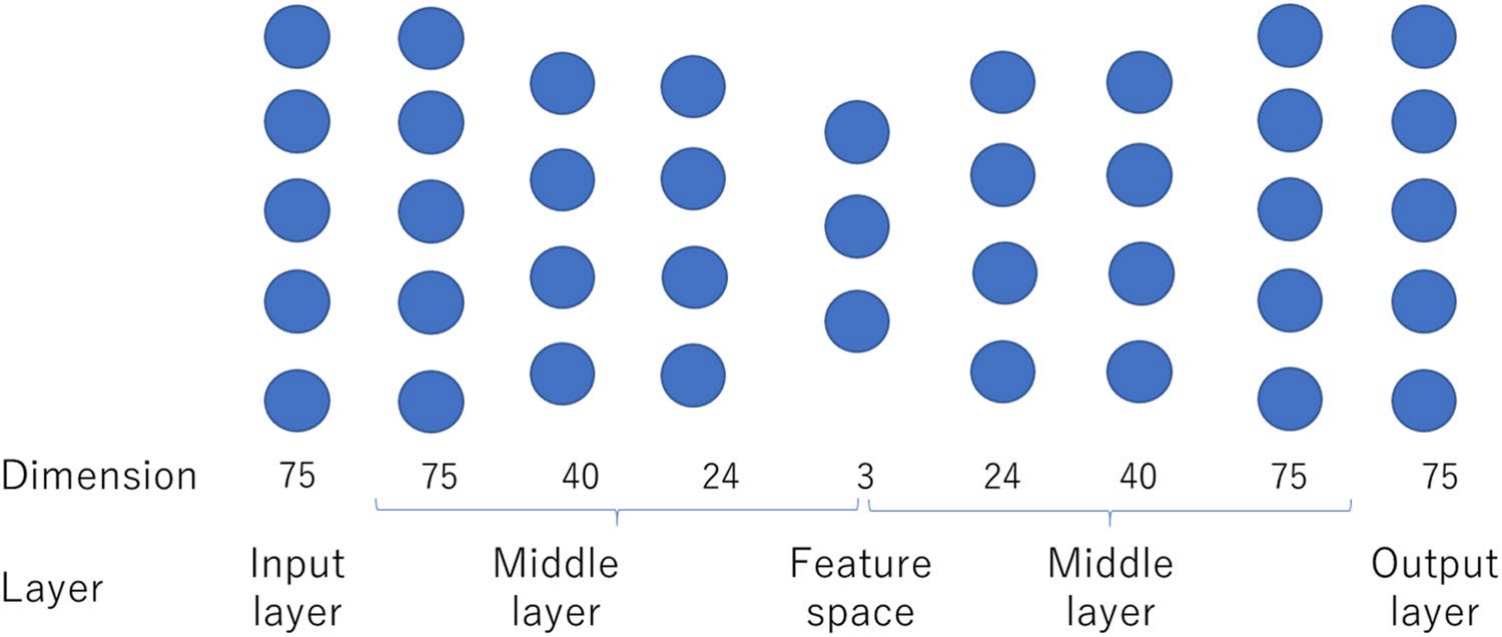
Neural network-encoder-decoder architecture for multiparameter analysis. The autoencoder consisted of two parts, an encoder and a decoder. The encoder comprised an input layer with 75 dimensions and four hidden layers with 75, 24, 40, and 75 nodes, respectively. The decoder had the reverse structure, specifically an output layer with 75 dimensions and four hidden layers with 3, 24, 40, and 75 nodes, respectively

We attempted to stratify SLE patients by training a neural network using clinical data from NDDID-J. An autoencoder was used as a learning architecture to analyze the features acquired by the neural network [13, 14]. Figure 1 shows the layer structure of the autoencoder and the number of its nodes.

The autoencoder consisted of two parts, an encoder and a decoder. The encoder comprised an input layer with 75 dimensions and four hidden layers with 75, 24, 40 and 75 nodes, respectively. The decoder had the reverse structure, specifically an output layer with 75 dimensions and four hidden layers with 3, 24, 40 and 75 nodes, respectively. The three-dimensional vectors were plotted as features on a scatter diagram.

A cross-entropy loss (MSELoss) function was used to calculate loss based on the input values of the encoder and the output values of the decoder. Adam was used as the optimization algorithm. Regarding the learning conditions, the number of training iterations per epoch was 60 and the number of epochs was one. The specifications of the personal computer used for learning were as follows: OS: Windows 10 Professional; CPU: AMD Ryzen Threadripper 3990X 64-core Processor 4.30 GHz; RAM: 256 GB; GPU: NVIDIA Quadro RTX 5000.

### Statistics

Data are described as median (interquartile range), mean, or count. The correlation between the presence or absence of kidney involvement and that of other clinical manifestations was analyzed using test statistics to determine the difference between population proportions. All manifestations are presented as dichotomous variables. If a statistic is positive, there is a positive correlation, and vice versa. Patients whose data were distributed within a given area determined by sliding a window or threshold on the two-dimensional (x, z) feature plane were included in the statistics calculations.

## Results

**Fig. 2:**
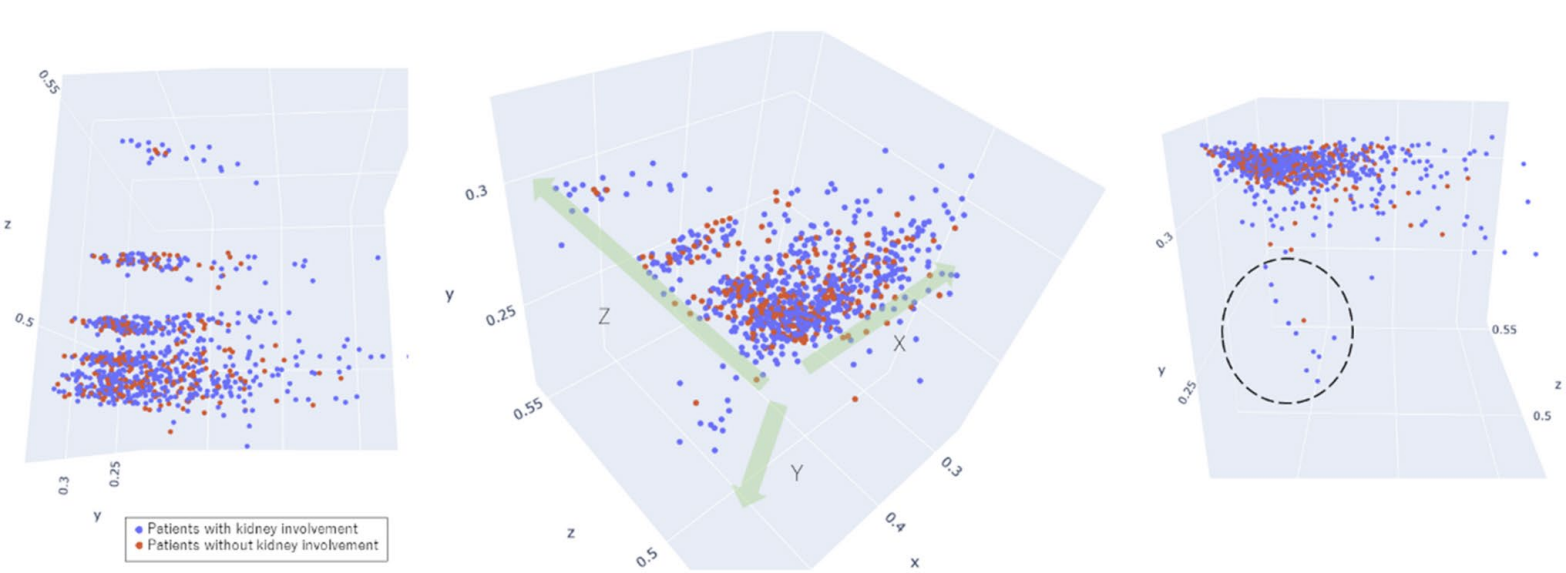
Scatter diagram of SLE patients in a feature space. A feature space obtained by machine learning is shown in a three-dimensional space and is viewed from three directions. Each dot on the scatter diagram indicates one patient. The coordinate axes x, y, and z were automatically defined by a learning model. In addition to these coordinate axes, we also defined feature vectors (indicated in green) as X, Y and Z. The feature vectors X and Y were obtained by converting the numbers of the coordinate axes x and y to the opposite signs (positive to negative, and negative to positive). The positive and negative numbers of feature vector Z correspond to those of the coordinate axis z. In the right diagram, patients with extremely high anti-dsDNA antibody titers (≥ 500, *n* = 49) had relatively low values on the y-axis (in the dashed circle)

The background demographics of the patients were shown in Supplementary Table S2. From among all SLE patients whose PCRs were newly registered (*n* = 4711), data from 1,655 patients without missing data were used to profile kidney involvement using machine learning. Patients with kidney involvement were mapped on the three-dimensional feature space obtained by the autoencoder (Fig. 2). Each dot on the scatter diagram indicates one patient. Blue and red dots show patients with and without kidney involvement, respectively, and 894 patients exhibited kidney involvement.

**Fig. 3:**
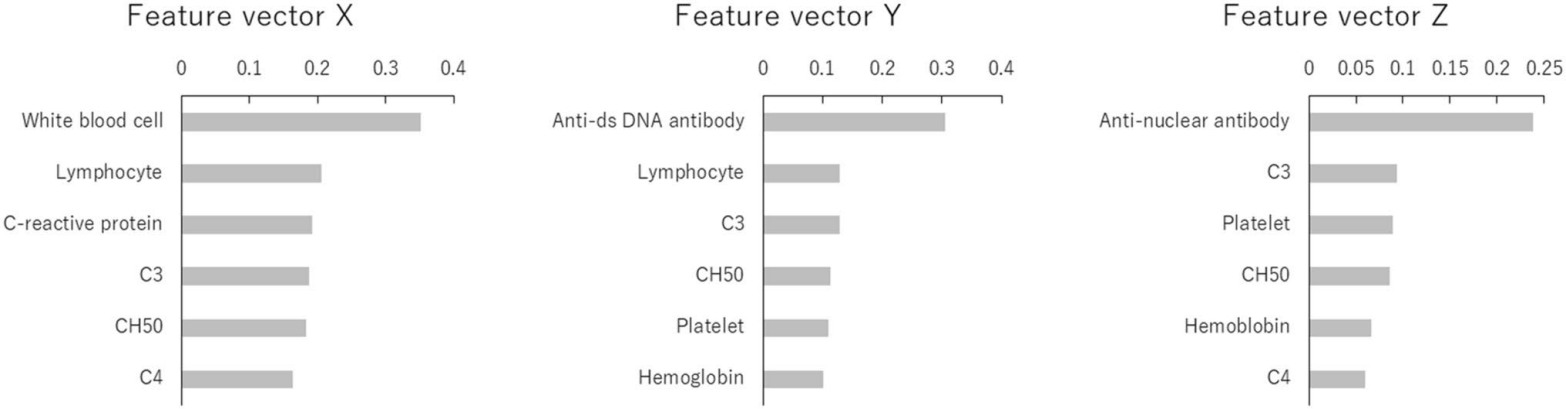
Contribution rates of input items for each feature vector. The items with the six highest contribution rates are shown for each feature vector, with contribution rates defined as the absolute values of the means of partial differentials for each sample. Partial differentials were calculated by defining the encoder outputs, i.e., the coordinates of the x, y and z axes, as the objective variables, and defining the input items as explanatory variables. This made it possible to qualitatively evaluate the relationship between the values of the input items and the coordinates in the three-dimensional feature space. Anti-dsDNA antibody, anti-double-stranded deoxyribonucleic acid antibody

For convenience, the coordinate axes were named x, y, and z. The encoder part of the autoencoder consisted of 75 input items that were used for learning. Output values can be considered to be functions of three-dimensional vectors that are coordinate values in the three-dimensional feature space. Moreover, coordinate values in this feature space can be considered to be functions of independent scalars. To analyze features, first-order partial differential values were calculated for patients with kidney involvement, assuming that the encoders were the functions of these three independent scalars. For each feature vector (X, Y, and Z, green arrows in Fig. 2), the absolute values of the means of first-order partial differential values were summarized as contribution rates (Fig. 3, Supplementary Table S3).

**Fig. 4:**
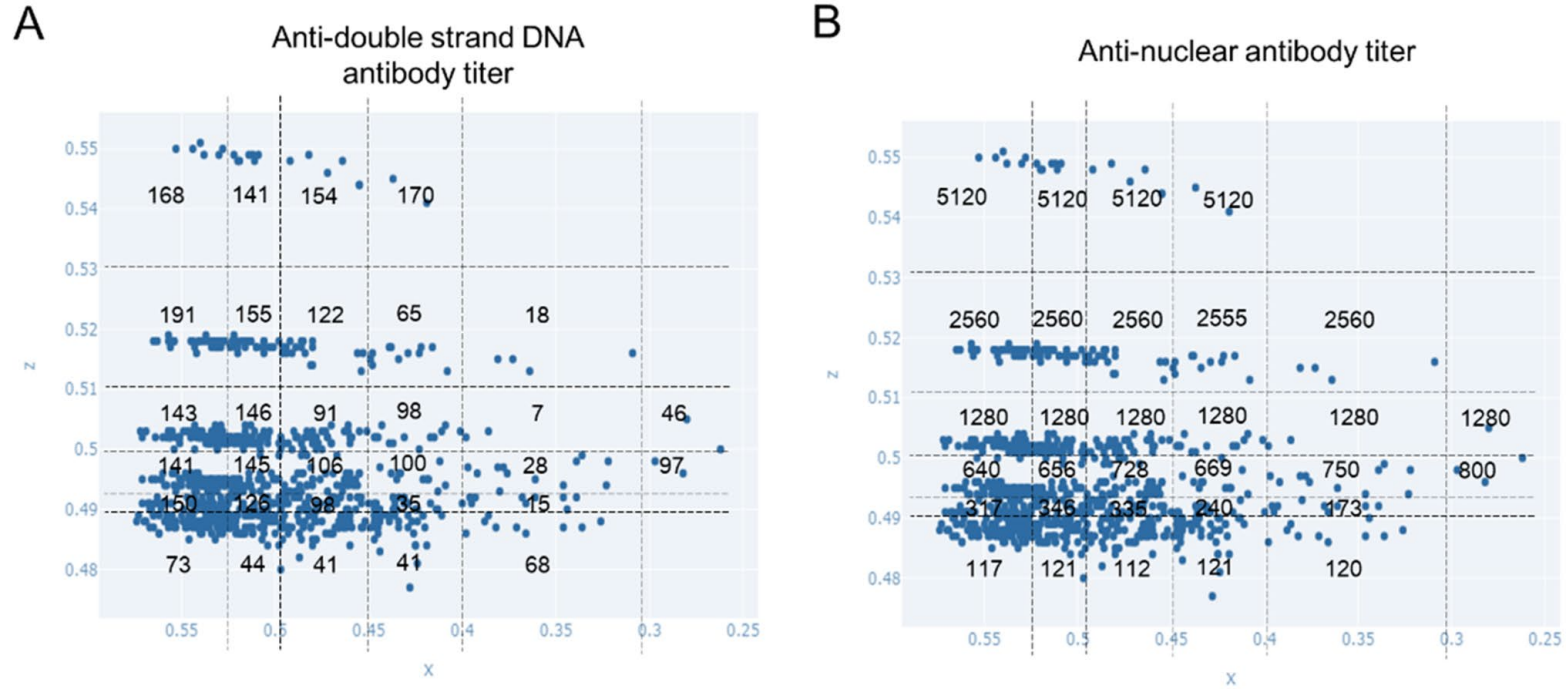
Antibody titers on the two-dimensional (x, z) feature plane. **A** Anti-double-stranded deoxyribonucleic acid antibody titer and **B** antinuclear antibody titer. Values, means. DNA, deoxyribonucleic acid

In the direction of feature vector X, increases in white blood cells, lymphocytes, and complement were observed. In the direction of feature vector Y, an increase in anti-dsDNA antibody titer was observed. In the direction of feature vector Z, an increase mainly in antinuclear antibody titer was observed. Although the anti-dsDNA antibody titer increased in the direction of feature vector Y, the absolute values of the partial differential of the anti-dsDNA antibody titer on the y-axis also increased in the direction of the x-axis (Supplementary Figure S1). Therefore, on the two-dimensional (x, z) feature plane, the anti-dsDNA antibody titer increased in the direction of the x-axis (Fig. 4A, Supplementary Table S4). Furthermore, based on the discussion of the contribution rates presented above, the discrete distribution on the z-axis links to the antinuclear antibody titer (Fig. 4B, Supplementary Table S5) and the continuous distribution on the x-axis links to the white blood cell count. Since the feature vectors can be explained on the two-dimensional (x, z) feature plane as described above, we decided to perform a detailed analysis using this plane. Although patients with extremely high anti-dsDNA antibody titers (≥ 500, *n* = 49) had relatively low values on the y-axis (i.e., within the black dashed circle on the right diagram in Fig. 2), for the sake of convenience we excluded them from the analysis using the two-dimensional (x, z) feature plane.

**Fig. 5:**
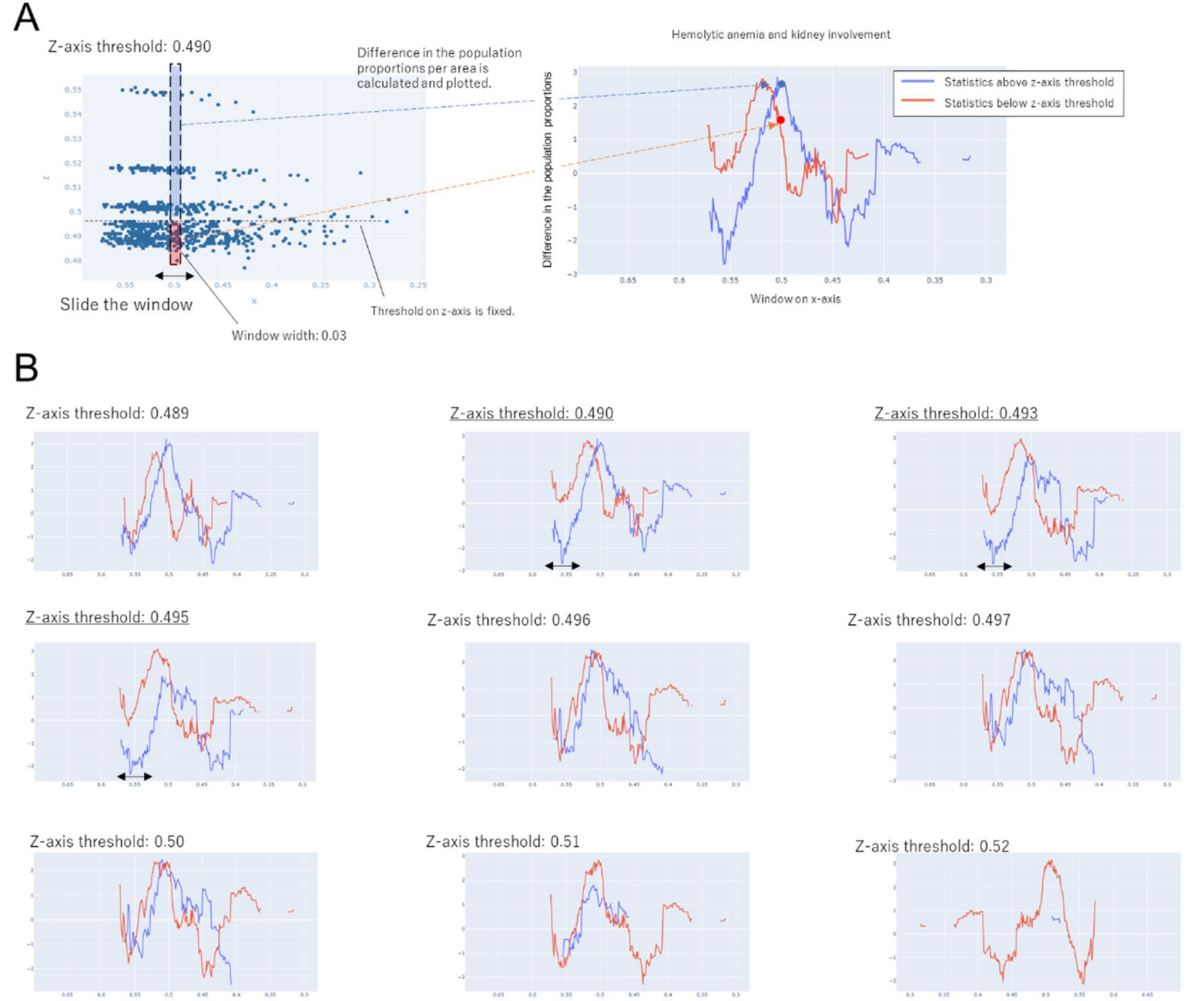
Changes in statistics between the presence or absence of kidney involvement and that of hemolytic anemia. **A** Methods for setting areas to calculate statistics on the two-dimensional (x, z) feature plane and for calculating statistics. (Left) Fixing the thresholds on the z-axis, the window was slid along the x-axis. Setting the window width to 0.03, two areas were determined (red and blue). (Right) The statistics of these areas were calculated and plotted on the vertical axis, and the position of the window was indicated on the horizontal (x) axis to visualize their changes. **B** For each of the eight z-axis thresholds, the correlation between the presence or absence of kidney involvement and that of hemolytic anemia is shown. When the threshold was 0.490, 0.493, or 0.495, there was a range of x-axis values (horizontal arrows) for which the statistics were significantly different between the red and blue lines (vertical arrows)

Using this plane, the correlation between the presence or absence of kidney involvement and that of other clinical manifestations was evaluated. For this purpose, data of patients that were distributed within a given area determined by sliding a window or threshold on the two-dimensional plane were analyzed. The dashed line and dashed box in Fig. 5A indicate the threshold and the window, respectively. As shown in Patterns 1 and 2, the window was continuously moved along the x and z axes to evaluate the difference between two given areas.

As shown in Pattern 1 in Fig. 5A, target areas were determined first, and then the statistics in the areas were calculated. Setting the window width to 0.03, two areas were determined (the red and blue areas on the left diagram in Fig. 5A) and the statistics of these areas were calculated. The horizontal and vertical axes of the graph show the starting point of the x-axis window and the statistics, respectively. Sliding the window in increments of 0.01 along the x-axis made it possible to comprehensively view the changes in the statistics (Fig. 5A). Setting the z-axis threshold to 0.489, 0.490, 0.493, 0.495, 0.496, 0.497, 0.500, or 0.510, the 0.03-wide window was slid along the x-axis. Statistics were calculated between the presence or absence of kidney involvement and that of other clinical manifestations. These statistics were plotted on the vertical axis, and the position of the window was indicated on the horizontal (x) axis to visualize their changes. The changes in the statistics between the presence or absence of kidney involvement and that of hemolytic anemia varied depending on the z-axis threshold (Fig. 5B). When the z-axis threshold was 0.490, 0.493, or 0.495 and the position of the window on the x-axis was around 0.55, the statistics differed between the area equal to or above the threshold and that below the threshold.

**Fig. 6:**
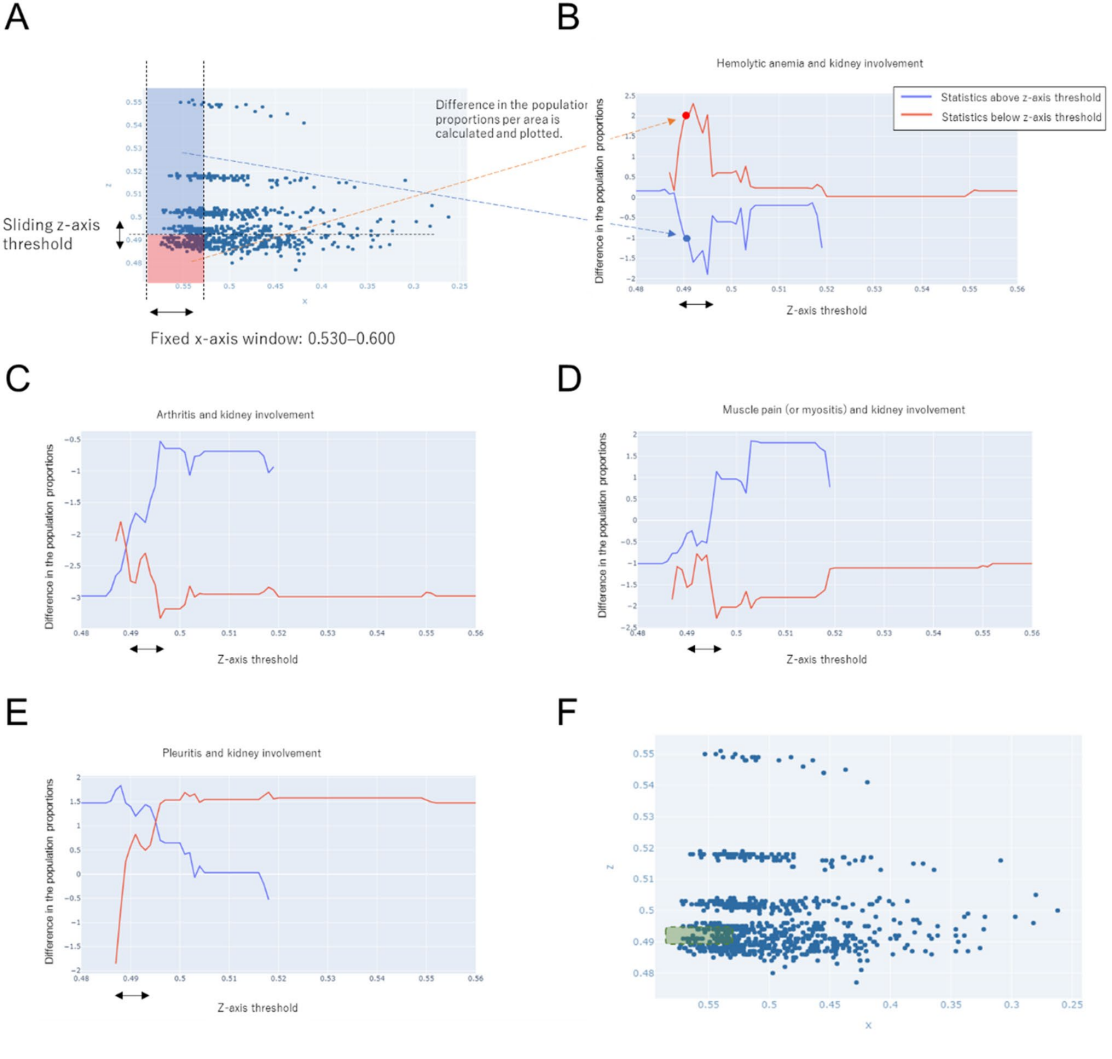
Correlation between the presence or absence of kidney involvement and that of arthritis, myalgia (myositis), or pleurisy. **A** Methods for setting areas and for calculating statistics. (Left) By fixing the thresholds on the x-axis and sliding the window along the z-axis, two areas were determined (red and blue). (Right) The statistics of these areas were calculated and plotted on the vertical axis, and the position of the window was indicated on the horizontal (z) axis to visualize their changes Statistics in the red area (left) correspond to the dot on the red line (right), and those in the blue area (left) correspond to the dot on the blue line (right). **B**–**E** The z-axis threshold was slid. Correlation between kidney involvement and **B** hemolytic anemia, **C** non-destructive arthritis (two or more lesions), **D** myalgia (myositis), or **E** pleurisy. **F** An area defined by an x-axis value of 0.530 or higher and a z-axis value of 0.490–0.495 represents patients with distinct features (green area in the diagram)

Further detailed analysis between kidney involvement and hemolytic anemia was performed to evaluate the dependency of the statistics on the z-axis. In this analysis, the statistics between the presence or absence of kidney involvement and that of hemolytic anemia in the area equal to or above the z-axis threshold and that below the z-axis threshold were calculated by setting the range of the x-axis to around 0.55 (range: 0.6–0.53) and changing the z-axis threshold according to the Pattern 2 in Fig. 6A. The thresholds of the z-axis and statistics were plotted on the horizontal and vertical axes, respectively (Fig. 6B). The graph showed that at z-axis thresholds of around 0.490–0.495, the relationship between kidney involvement and hemolytic anemia was symmetrical with respect to the threshold. In particular, it showed that there was a high likelihood that kidney involvement was positively correlated with hemolytic anemia in the area below the z-axis threshold.

The correlation between kidney involvement and inflammatory manifestations was also analyzed. There were significant changes in the statistics of arthritis, myalgia (myositis), and pleurisy at z-axis thresholds of around 0.490–0.495 (Fig. 6C, D). In the area above the z-axis threshold, the correlation between arthritis or myalgia (myositis) and kidney involvement was less negative or became positive at z-axis thresholds of around 0.490–0.495. This correlation seemed to be the reverse of that between hemolytic anemia and kidney involvement. The correlation between pleurisy and kidney involvement changed from negative to positive at z-axis thresholds of around 0.487–0.495 in the area below the z-axis threshold, and a negative correlation was observed only at z-axis thresholds below 0.489 (Fig. 6E).

These findings indicated that there was a negative correlation between inflammatory manifestations and kidney involvement in the areas at z-axis thresholds of less than 0.495 and that the correlation significantly changed when the threshold was increased to 0.495. Therefore, the nature of the relationship between inflammatory manifestations and kidney involvement may be different from that between hemolytic anemia and kidney involvement, which was positive at thresholds equal to or below 0.495.

These results showed that patients whose data were included in the area defined by an x-axis value of approximately 0.53–0.60 and a z-axis value of approximately 0.490–0.495 (green area in Fig. 6F) demonstrated a positive correlation between kidney involvement and hemolytic anemia and a negative correlation between kidney involvement and some inflammatory manifestations.

## Discussion

Using machine learning based on NDDID-J clinical data of SLE patients, this study revealed that the distributions of SLE features depended mainly on the antinuclear antibody titer, anti-ds-DNA antibody titer, and white blood cell count. Although the features of patients with and without kidney involvement largely overlapped, a subgroup of SLE patients with kidney involvement had distinct features in terms of other clinical manifestations. These findings indicated the complexity of SLE manifestations and that there were distinct features of kidney involvement specific to each of the divided feature spaces.

The data of SLE patients were compressed in a feature space in which the anti-dsDNA antibody titer, antinuclear antibody titer, or white blood cell count contributed significantly to distinguishing between different patient subgroups. Since these parameters are associated with the diagnosis and severity of SLE, patient manifestations may have been reflected in the division of the feature space. However, this analysis alone was insufficient to identify manifestations specific to certain patient subgroups, probably because of the diversity of SLE manifestations. All manifestations were complicated with kidney involvement, indicating that kidney involvement is diverse and may have various pathophysiologies. SLE-related kidney involvement may be a direct manifestation of SLE in some patients and a complication of SLE in others. The presence of kidney involvement that was undetected using PCRs may have obscured the characteristics.

Detailed analysis identified a subgroup of patients whose antinuclear antibody titer was low and whose anti-dsDNA antibody titer was high. Patients in this population had hemolytic anemia but no inflammatory manifestations, such as arthralgia or myalgia. A close association between kidney involvement and hemolytic anemia has been reported [15, 16]. Anti-dsDNA antibodies were also associated with these manifestations [15, 16], where pathogenic antibodies are crucial in disease development [17]. Conversely, systemic inflammatory manifestations, such as malaise, fatigue, fever, arthralgia, and myalgia, are caused by type I interferon (IFN) signaling [18]. These inflammatory manifestations responded well to the treatment with an anti-type I IFN receptor antibody, anifrolumab, while renal manifestations did not [19]. Thereby, the pathophysiological etiologies of kidney involvement with hemolytic anemia are different from that with inflammatory manifestations. Further studies will clarify the causality between hemolytic anemia and kidney involvement and identify associated biomarkers.

The machine learning used in this study has recently begun to be applied to clinical research [9, 12]. Although supervised machine learning may increase diagnostic precision [20], it provides no further benefits, particularly for intractable diseases with unknown causes and complicated manifestations. Since the validity of current disease classifications is questionable, diseases must be classified based on objective findings obtained by unsupervised machine learning. The method used in this study is needed for patient stratification which will make it possible to determine treatment strategies in clinical settings and to develop novel therapies. The use of this approach in combination with new biomarkers such as d-amino acids should increase our understanding of intractable diseases [21, 22].

This study has several limitations. It was cross-sectional in nature. The profiling based on longitudinal changes in clinical conditions may clarify the parameters associated with newly-identified subgroups. Patients without missing data were excluded from the analysis, suggesting a possibility of selection bias. Although data were collected from PCR forms and their accuracy depended largely on those who completed the forms, we evaluated the validity of the data to the greatest extent possible. The validity of the analysis itself should be assessed in further studies using data from other cohorts.

In summary, this study attempted to objectively profile various manifestations of SLE using unsupervised machine learning. Many SLE manifestations are accompanied by kidney involvement, whereas some subgroups of patients with SLE-related kidney involvement have distinct features. Some of the clinical manifestations were associated with kidney involvement. We expect that this study will serve as a foundation for further research on the complicated manifestations of SLE and the mechanism of onset of SLE-related kidney involvement.

## Supplementary Information

Below is the link to the electronic supplementary material.

Supplementary file1 (PDF 542 KB)

## Data Availability

Data availability is under the strict control of the working group on data provision of the Ministry of Health, Labour, and Welfare, Japan.
