# Supplementary material for: Profiling of kidney involvement in systemic lupus erythematosus by deep learning using the National Database of Designated Incurable Diseases of Japan
Source: Clin Exp Nephrol. 2023 Mar 16;27(6):519–27. doi: 10.1007/s10157-023-02337-x (PMC10191896; doi:10.1007/s10157-023-02337-x)
Supplement: Supplementary file 1 — Supplementary file1 (PDF 542 KB) [file 10157_2023_2337_MOESM1_ESM.pdf]

Supplementary information

This PDF file includes:

Supplementary Tables S1–S5
Supplementary Figures S1

Supplementary Table 1. Variables used in this study.

Input variables									
Nursing care certification	{1, 2, 3}	Muscle pain or myositis	{1, 2, 3}	Cranial nerve disorder	{1, 2, 3}	Cerebral infarction	{1, 2, 3}	Creatinine	{Continous value}
Degree of movement	{1, 2, 3}	Muscle weakness	{1, 2, 3}	Mononeuritis multiplex	{1, 2, 3}	Compression fracture	{1, 2, 3}	C3	{Continous value}
Personal care	{1, 2, 3}	Pericarditis	{1, 2, 3}	Disturbance of consciousness	{1, 2, 3}	Osteonecrosis	{1, 2, 3}	C4	{Continous value}
Usual activities	{1, 2, 3}	Pulmonary hypertension	{1, 2, 3}	Cerebrovascular accident	{1, 2, 3}	Disseminated intravascular coagulation	{1, 2, 3}	CH50	{Continous value}
Pain / discomfort	{1, 2, 3}	Interstitial pneumonia	{1, 2, 3}	Spinal cord injury	{1, 2, 3}	Qualitative protein	{1, 2, 3}	Anti-nuclear antibody	{20, 40, 60, 80, 100, 126, 160, 180, 320, 640, 1250, 1260, 1280, 2500, 2560, 5120}
Anxiety / depression	{1, 2, 3}	Pulmonary hemorrhage	{1, 2, 3}	Aseptic meningitis	{1, 2, 3}	Qualitative occult blood	{1, 2, 3}	Anti-double-stranded DNA antibody titer	{Continous value}
A fever of 38 degrees or higher that continues for more than 2 weeks	{1, 2, 3}	Pulmonary infarction	{1, 2, 3}	Visual impairment	{1, 2, 3}	Red blood cells sediment	{1, 2, 3}	Elevated anti-DNA antibodies	{1, 2, 3}
Facial erythema	{1, 2, 3}	Pleurisy	{1, 2, 3}	Lupus headache	{1, 2, 3}	Granular cast sediment	{1, 2, 3}	Anti-U1-RNP antibody	{1, 2, 3}
Discoid rash	{1, 2, 3}	Rapidly progressive glomerulonephritis	{1, 2, 3}	Hemolytic anemia	{1, 2, 3}	Hematuria	{1, 2, 3}	Anti-Sm antibody	{1, 2, 3}
Photosensitivity	{1, 2, 3}	Nephrotic syndrome	{1, 2, 3}	Vasculitis	{1, 2, 3}	Pyuria	{1, 2, 3}	Anti-SS-A antibody	{1, 2, 3}
Cold extremities, numbness, Raynaud's phenomenon	{1, 2, 3}	Aacute renal failure	{1, 2, 3}	Malignant tumor	{1, 2, 3}	White blood cell count	{Continous value}	Anti-SS-B antibody	{1, 2, 3}
Hair loss	{1, 2, 3}	Chronic renal failure	{1, 2, 3}	Infection	{1, 2, 3}	Hemoglobin	{Continous value}	Serum syphilis reaction (biological false positive)	{1, 2, 3}
Gastrointestinal ulcer	{1, 2, 3}	Seizures	{1, 2, 3}	Diabetes	{1, 2, 3}	Platelet	{Continous value}	anticardiolipin antibody	{1, 2, 3}
Oral or nasal ulcers	{1, 2, 3}	Psychiatric symptoms	{1, 2, 3}	High blood pressure	{1, 2, 3}	Lymphocyte	{Continous value}	Anti-CLβ2GP1 antibody	{1, 2, 3}
Arthritis (nondestructive, ≥2 sites)	{1, 2, 3}	Organic brain disorder	{1, 2, 3}	Myocardial infarction	{1, 2, 3}	C-reactive protein	{Continous value}	Lupus anticoagulant	{1, 2, 3}

Supplementary Table S2. Background demographics of the patients.

	Total patients n = 1,655			Patients with kidney involvement n = 894		
Continuous variables	Median	25% quartile	75% quartile	Median	25% quartile	75% quartile
White blood cell (/μL)	3900	2800	5900	3725	2700	5800
Hb (g/dL)	11.2	9.7	12.7	10.9	9.2	12.375
Platelet (x 10^4/μL)	18.9	12.2	24.85	18	11.2	24.5
Lymphocyte (/mm^3)	902	606.5	1294	870	598.5	1230
C reactive protein (mg/dL)	0.3	0.085	1.37	0.3	0.0925	1.475
Serum creatinine (mg/dL9	0.63	0.53	0.82	0.675	0.55	0.94
C3 (mg/dL)	63	41.15	86.5	57	36.7	82
C4 (mg/dL)	10	5	17	8.7	4.3	15
CH50 (U/dL)	27.4	14	40.85	24	12	37.725
Anti-nuclear antibody titer	640	160	1280	640	160	1280
Anti-ds DNA antibody titer	28	10	120.05	38.05	10	210

Categorical variables	1	2	3	1	2	3
Nursing care certification	34	17	1604	16	10	868
Degree of movement	1069	539	47	568	298	28
Personal care	1196	396	63	642	216	36
Usual activities	917	675	63	496	360	38
Pain / discomfort	719	809	127	398	424	72
Anxiety / depression	986	589	80	530	314	50
A fever of 38 degrees or higher that continues for more than 2 weeks	525	1085	45	294	576	24
Facial erythema	761	888	6	401	491	2
Discoid rash	336	1311	8	165	726	3
Photosensitivity	606	1013	36	302	573	19
Cold extremities, numbness, Raynaud's phenomenon	617	1028	10	298	591	5
Hair loss	495	1141	19	247	638	9
Gastrointestinal ulcer	55	1346	254	25	739	130
Oral or nasal ulcers	398	1238	19	223	661	10
Arthritis (nondestructive, ≥2 sites)	1091	563	1	539	354	1
Muscle pain or myositis	454	1188	13	228	660	6
Muscle weakness	332	1305	18	177	708	9
Pericarditis	246	1362	47	148	727	19
Pulmonary hypertension	42	1449	164	16	800	78
Interstitial pneumonia	135	1503	17	71	811	12
Pulmonary hemorrhage	27	1613	15	15	873	6
Pulmonary infarction	17	1604	34	12	869	13
Pleuritis	283	1353	19	165	718	11
Rapidly progressive glomerulonephritis	140	1485	30	123	753	18
Nephrotic syndrome	272	1375	8	244	646	4
Aacute renal failure	129	1513	13	115	773	6
Chronic renal failure	153	1492	10	114	770	10

(Continues)

Seizures	57	1595	3	26	867	1
Psychiatric symptoms	135	1507	13	65	822	7
Organic brain disorder	48	1591	16	31	854	9
Cranial nerve disorder	35	1614	6	16	876	2
Mononeuritis multiplex	68	1569	18	32	849	13
Disturbance of consciousness	71	1581	3	40	851	3
Cerebrovascular accident	104	1531	20	59	826	9
Spinal cord injury	9	1637	9	3	888	3
Aseptic meningitis	37	1600	18	15	871	8
Visual impairment	49	1589	17	26	860	8
Lupus headache	149	1481	25	71	808	15
Hemolytic anemia	212	1419	24	120	763	11
Vasculitis	126	1484	45	72	796	26
Malignant tumor	29	1573	53	18	843	33
Infection	118	1521	16	75	812	7
Diabetes	143	1511	1	95	798	1
High blood pressure	323	1330	2	217	675	2
Myocardial infarction	15	1638	2	10	882	2
Cerebral infarction	94	1551	10	50	838	6
Compression fracture	47	1603	5	22	871	1
Osteonecrosis	38	1606	11	14	877	3
Disseminated intravascular coagulation	16	1635	4	11	882	1
Qualitative protein	868	778	9	606	288	0
Qualitative occult blood	670	974	11	493	401	0
Red blood cells sediment	616	999	40	476	409	9
Granular cast sediment	422	1182	51	375	504	15
Hematuria	390	1246	19	316	574	4
Pyuria	262	1373	20	200	690	4
Elevated anti-DNA antibodies	1127	380	148	616	193	85
Anti-U1-RNP antibody	503	1152	0	358	536	0
Anti-Sm antibody	909	746	0	283	611	0
Anti-SS-A antibody	270	1385	0	496	398	0
Anti-SS-B antibody	122	1207	326	144	750	0
Serum syphilis reaction (biological false positive)	388	1267	0	71	680	143
anticardiolipin antibody	223	1432	0	231	663	0
Anti-CLβ2GP1 antibody	278	1377	0	124	770	0
Lupus anticoagulant	278	1377	0	154	740	0

Data, median (range) or count.

Supplementary Table S3. Means of first-order partial differential values for each feature vector.

Feature vector	White blood cell	Hemoglobin	Platelet	Lymphocyte	C−reactive protein	Creatinine	C3	C4	CH50	Anti−nuclear antibody	Anti−ds DNA antibody	Anti−DNA antibody
X	−0.35182	−0.15588	−0.15748	−0.20569	−0.19229	−0.07798	−0.18722	−0.16346	−0.18231	−0.05507	−0.13679	−0.07116
Y	0.014145	−0.10085	−0.10945	−0.12896	0.075542	−0.02225	−0.1287	−0.08133	−0.11243	−0.08745	−0.30514	−0.03858
Z	0.005763	0.066288	0.089267	−0.04619	−0.00432	0.013037	0.09402	0.059518	0.085403	0.238002	0.057184	0.02722

Supplementary Table S4. Anti-dsDNA antibody titers on the two-dimensional (x, z) feature plane.

Mean

167.8	140.525	154.45	169.6	nan	nan
191.2593	154.5962	121.5368	64.55455	17.825	nan
143.4217	145.9036	90.57805	97.91538	6.95	46.05
140.6935	144.622	106.01	100.0455	27.98333	96.5
149.8196	125.7655	98.44762	34.615	15.27778	nan
72.57971	44.48308	41.12034	40.77576	68.2	nan

Standard deviation

210.3786	173.0942	171.9515	122.1881	nan	nan
159.4068	154.8605	153.6	122.837	18.56491	nan
153.6104	150.2964	119.6988	139.1988	4.313351	63.5689
152.0417	139.1509	134.9732	122.4473	32.16675	19.09188
155.4981	141.7536	138.1594	88.51956	23.67297	nan
108.7276	52.30433	71.5027	71.25942	125.3113	nan

The two-dimensional (x, z) feature plane was divided into 6x6 cells to generate 36 areas. The means and standard deviations for each of these 36 areas are given in the tables (means, upper table; standard deviations, lower table).

Supplementary Table S5. Antinuclear antibody titers on the two-dimensional (x, z) feature plane.

Mean

5120	5120	5120	5120	-	-
2560	2560	2560	2554.545	2560	-
1279.71	1280	1279.512	1280	1280	1280
640	655.6098	728	669.0909	750	800
316.8627	345.5172	335.2381	240	173.3333	-
117.1014	121.3231	111.5068	121	120	-

Standard deviation

0	0	0	0	-	-
0	0	0	18.09068	0	-
2.407717	0	3.123475	0	0	0
0	99.95121	240.205	224.2077	432.5401	678.8225
59.78261	109.6614	126.4764	139.1705	140	-
72.70208	80.92456	99.3976	89.36162	91.65151	-

The two-dimensional (x, z) feature plane was divided into 6x6 cells to generate 36 areas. The means and standard deviations for each of these 36 areas are given in the tables (means, upper table; standard deviations, lower table).

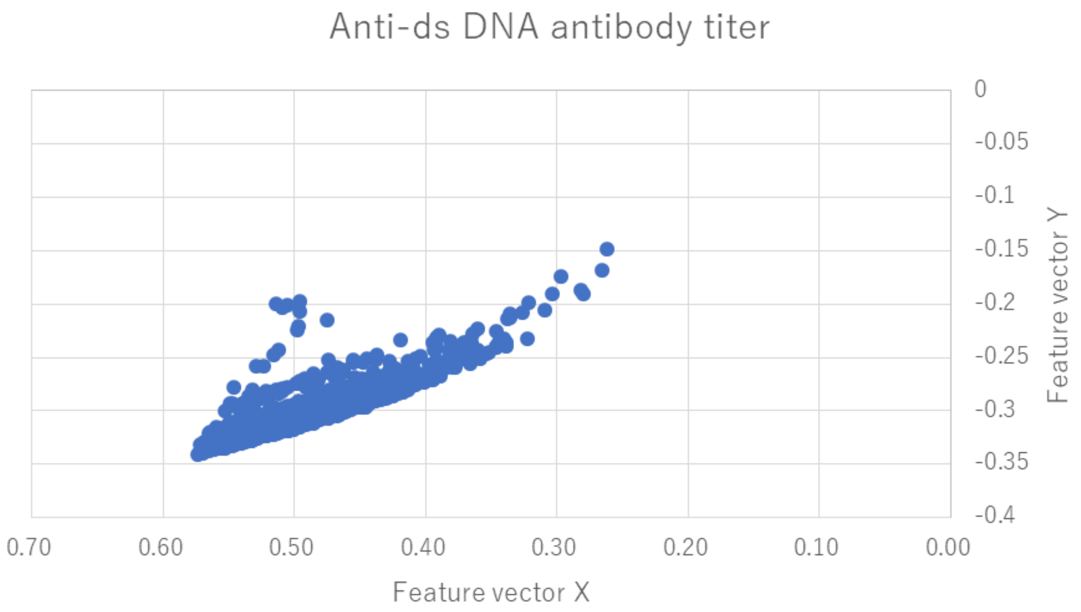

Supplementary Figure S1. Anti-dsDNA antibody titers on the two-dimensional (x, z) feature plane. While the anti-dsDNA antibody titer increases as the y-axis value decreases, the rate of increase rises as the x-axis value increases. Therefore, on the two-dimensional (x, z) feature plane, it appears that the anti-dsDNA antibody titer increases in the direction of x-axis.
